# Differential role of dose and environment in initiating and intensifying neurotoxicity caused by MDMA in rats

**DOI:** 10.1186/s40360-019-0326-6

**Published:** 2019-08-05

**Authors:** Ibrahim M. Shokry, Connor J. Shields, John J. Callanan, Zhiyuan Ma, Rui Tao

**Affiliations:** 1 Department of Biomedical Science, Charles E. Schmidt College of Medicine, Florida Atlantic University, 777 Glades Road, Boca Raton, FL 33431 USA; 2 Ross University School of Veterinary Medicine, Basseterre, Saint Kitts and Nevis

**Keywords:** MDMA toxicity, Serotonin syndrome, Serotonergic injury, Hypothermia, Hyperthermia, Microdialysis, EEG, Environment, Initiation, Intensification

## Abstract

**Background:**

MDMA causes serotonin (5-HT) syndrome immediately after administration and serotonergic injury in a few days or weeks. However, a serotonin syndrome is not always followed by serotonergic injury, indicating different mechanisms responsible for two adverse effects. The goal of present study was to determine causes for two adverse events and further test that dose and environment have a differential role in initiating and intensifying MDMA neurotoxicity.

**Methods:**

Initiation and intensification were examined by comparing neurotoxic effects of a high-dose (10 mg/kg × 3 at 2 h intervals) with a low-dose (2 mg/kg × 3) under controlled-environmental conditions. Initiation of a serotonin syndrome was estimated by measuring extracellular 5-HT, body-core temperature, electroencephalogram and MDMA concentrations in the cerebrospinal fluid, while intensification determined in rats examined under modified environment. Initiation and intensification of the serotonergic injury were assessed in rats by measuring tissue 5-HT content, SERT density and functional integrity of serotonergic retrograde transportation.

**Results:**

Both low- and high-dose could cause increases in extracellular 5-HT to elicit a serotonin syndrome at the same intensity. Modification of environmental conditions, which had no impact on MDMA-elicited increases in 5-HT levels, markedly intensified the syndrome intensity. Although either dose would cause the severe syndrome under modified environments, only the high-dose that resulted in high MDMA concentrations in the brain could cause serotonergic injury.

**Conclusion:**

Our results reveal that extracellular 5-HT is the cause of a syndrome and activity of postsynaptic receptors critical for the course of syndrome intensification. Although the high-dose has the potential to initiate serotonergic injury due to high MDMA concentrations present in the brain, whether an injury is observed depends upon the drug environment via the levels of reactive oxygen species generated. This suggests that brain MDMA concentration is the determinant in the injury initiation while reactive oxygen species generation associated with the injury intensification. It is concluded that the two adverse events utilize distinctly different mediating molecules during the toxic initiation and intensification.

**Electronic supplementary material:**

The online version of this article (10.1186/s40360-019-0326-6) contains supplementary material, which is available to authorized users.

## Background

3,4-Methylenedioxy-N-methylamphetamine (MDMA, ‘Ecstasy’) is a serotonin (5-hydroxytryptamine; 5-HT) releaser that increases extracellular 5-HT in the brain immediately after administration [1–3]. Excessive 5-HT levels are, to some extent, neurotoxic, resulting in a wide array of symptoms called serotonin syndrome [4]. In rodents, occurrence of a syndrome behaviorally showing hyperlocomotor activity, head shakes, flat body posture, shivering and salivation [5, 6] is not always notable or reliably used to assess the degree of a syndrome. Other behavioral abnormalities such as forepaw treading, hind limb abduction, and Straub tail, which typically occur in the syndrome caused by 5-hydrotryptophan in combination with a monoamine oxidase inhibitor [7, 8], are relatively rare in the MDMA-elicited serotonin syndrome. Despite difficulty in behavior measurement, the syndrome intensity can be instrumentally estimated by measuring changes in body-core temperature (*T*_cor_), as seen that hypothermia in the mild syndrome and hyperthermia in the severe syndrome [4]. Hypothermia reflects a strong mobilization of natural neuroprotective mechanisms against neurotoxicity [9], while hyperthermia indicates serious neurotoxicity overwhelmed the neuroprotection. However, hyperthermia is not elicited unless MDMA administration takes place in a modified environment, e.g., warmer ambient temperature (> 24 °C; [10]).

In addition to a syndrome, MDMA administration may also cause another toxic effect on the serotonergic system in a few days to weeks, showing a reduction in tissue 5-HT content and SERT density collectively called serotonergic injury. However, the relationship between the syndrome intensity and serotonergic injury remains elusive. Some reports demonstrated that a delayed injury in the brain was found not only in animals previously with hyperthermia but also from those with hypothermia [11], implicating that the syndrome intensity has no effect on serotonergic injury. Others demonstrated that the injury did not occur unless animals had had hyperthermia previously [10, 12, 13], arguing that serotonergic injury occurs only with the severe syndrome. The causes for these conflicting observations are unknown, but likely suggest that mechanisms for initiating serotonergic injury differ from those for serotonin syndrome. Drug dose levels are known to be critical for MDMA toxicity, and thus the majority of investigations prior to 2000s were conducted with the dose as high as 10 mg/kg (for instance, [14, 15]). Recently, an accumulating body of evidence has demonstrated that MDMA toxicity in rats should occur at much lower doses (see details reviewed by Baumann et al. [16]). Whether the high- and low-dose have different effects on the development of serotonin syndrome and serotonergic injury still remains elusive.

Our previous study demonstrated that the mechanistic course of a serotonin syndrome involved at least two distinct processes: syndrome initiation and intensification [3]. Specifically, the syndrome is initiated by excessive 5-HT in the brain and intensified by activation of postsynaptic 5-HT_2A_ receptors. In addition, drug environments are found to be critical for intensifying activity of 5-HT_2A_ receptors in accord with the syndrome development from the mild to severe levels. The aim of the present study was to test that MDMA-elicited serotonergic injury also follows a course of initiation and intensification, but its mediating molecules differ from those for serotonin syndrome.

## Methods

### Animals

A total of 176 male Sprague-Dawley rats weighing 275 -300 g were purchased from Charles River laboratories (Raleigh, NC, USA), and kept in standard Plexiglas cages (3 rats/cage) under a 12:12 h light-dark cycle in a temperature (22 ± 1 °C) and humidity (40–70%)-controlled environment. Food and water were available at all times. Animal care and experiments were according to the National Institute of Health Guide for the Care and Use of Laboratory Animals, and approved by the Florida Atlantic University and Ross University School of Veterinary Medicine Instructional Animal Care and Use Committee (IACUC).

### MDMA binge doses and environmental conditions

Previous studies show that serotonin syndrome was not elicited unless serotonergic drugs were systemically administered [17]. In the present study, we decided to utilize the intraperitoneal (i.p.) route to elicit serotonin syndrome. It has been suggested that 2.5 mg/kg, s.c., was closely similar to humans receiving 1.3–1.6 mg/kg p.o. MDMA in laboratory settings [18]. Because of relatively high efficacy with the intraperitoneal route, we reduced the low dose (LD) to 2 mg/kg in the present study. It has been suggested that the high dose (HD) relevant to human use is in a range of 7.5-10 mg/kg [12, 16], and thus we decided to use 10 mg/kg as HD for this study. To avoid a life-threatening syndrome, the binge given to rats was limited to 3 doses at 2 h intervals. (±)-MDMA was obtained from NIDA (Research Triangle Park, NC, USA.).

Animal experiments were conducted in a temperature-controlled chamber installed with a treadmill for physical exercise, Raturn (BASi; West Lafayette, IN, USA) for microdialysis or electroencephalogram (EEG) recording. Animals’ health was monitored throughout the experiments. The normal environment was defined as chamber temperature set at 22 °C (± 1 °C) in which animals were freely behaving (Additional file 1: Figure S1).

To test the role of environment in MDMA toxicity, temperature in the chamber was modified according to a drug dose examined in rats. Previous studies demonstrate that a small increase in the chamber temperature was sufficient to intensify the effects of a high-dose [12, 15]. Thus, rats with the high dose were examined in the chamber temperature at 26 °C (± 1 °C). However, temperature alone up to 32 °C (± 1 °C) could not markedly intensify effects of a low-dose [3], and thus a treadmill for physical activity was added for testing the rats administered the low dose. This design is in line with the drug environment for human users at rave-parties involving rigorous dancing behavior.

### Measures for syndrome initiation and intensification

Experiment 1 was designed to examine the syndrome initiation and thus the tests were conducted under a normal environmental condition. Experiment 2 was designed to determine changes in the syndrome intensity and therefore, experiments were performed under the modified environment [3]. Animals were placed in a temperature-controlled chamber for habitation at least 2 h prior to test initiation or intensification. After basal (pre-drug) collection, animals were administered with either low binge dose (LD; 2 mg/kg × 3 at 2 h intervals) or high binge dose (HD; 10 mg/kg × 3 at 2 h intervals). Serotonin syndrome and its intensity were instrumentally estimated by measuring changes in extracellular 5-HT, electroencephalogram (EEG) activity and body-core temperature (*T*_cor_). MDMA concentrations in the plasma and cerebrospinal fluid (CSF) were also determined. Details of four instrumental measures are as follows.

#### Microdialysis

Extracellular brain 5-HT was assessed using microdialysis as previously described in detail [3]. Briefly, a guide cannula (coordinates: AP + 3.2 mm relative to bregma, ML ±0.7 mm relative to midline, DV − 2.0 relative to dura) was implanted under anesthesia (80 mg/kg ketamine in combination of 4 mg/kg xylazine), 7 days prior to brain microdialysis. A microdialysis probe (exchange surface 2.5 mm in length, 0.2 mm in diameter, molecular cut-off 13 kD) was inserted through the guide into the prefrontal cortex (FCx; coordinates for the membrane tip: AP + 3.2 mm relative to bregma, ML ±0.7 mm relative to midline, DV − 4.5 mm relative to dura). The probe was perfused with the artificial cerebrospinal fluid (aCSF; 140 mM NaCl, 3.0 mM KCl, 1.5 mM CaCl_2_, 1.0 mM MgCl_2_, 0.25 mM NaH_2_PO_4_, and 1.0 mM Na_2_HPO_4_; pH 7.4) at a rate of 1.0 μl/min. Samples were collected at 15 min intervals, and analyzed by high-performance liquid chromatography with electrochemical detection (HPLC-EC; HTEC-500, Eicom, Japan). 5-HT was separated through a mobile phase (0.1 M phosphate buffer at pH 6.0, 500 mg/L 1-decanesulfonic acid, 50 mg/L EDTA, and 1% methanol) at a rate of 0.50 ml/min. Changes in 5-HT were expressed as mean (± s.e.m) of a fold increase above baseline.

#### Body-core temperature (*T*_cor_)

Thermoprobe consisting of a sensor, a cable (2.1 mm in diameter) and a digital meter were purchased from Measurement Specialties Inc. (MEAS 402, Dayton, OH, USA). The sensor was placed in the colon inserted to a depth of 9.0 mm from the anus. The cable connecting to the sensor was secured with a paper tape to the rat tail throughout an experiment, and thereby there was no physical stress or contact to the rest of data collection. Temperature readings on the meter were manually recorded at 15 min intervals. The mean of 4 consecutive measurements prior to drug was obtained as a baseline. Changes in *T*_*cor*_ from the baseline were expressed as mean (± s.e.m).

#### EEG

Brain activity was evaluated with EEG recording as described previously [6]. Positive, negative and reference electrodes were anchored on the skull over the left hemispheric FCx (AP + 2.0 mm relative to the bregma, ML 2.0 mm relative to the midline), the right hemispheric parietal cortex (AP − 7.0 mm relative to the bregma, ML 2.0 mm), and the left hemispheric parietal cortex, respectively. Digital data was filtered (low-pass filter set to 30 Hz and high-pass filter to 0.5 Hz) and continuously sampled by Chart 7 software (ADInstruments, Milford, U.S.A). EEG waves were analyzed off-line, and expressed as power spectral density (PSD; μV^2^/Hz). The baseline was the mean PSD levels before drug administration. Changes in EEG activity were normalized as the percentage changes relative to a respective PSD baseline at 0.25 Hz resolution.

#### MDMA assay

MDMA concentrations in the blood plasma and cerebrospinal fluid (CSF) were determined, 30 min after each administration. Blood was obtained from the tail vein and procedures to process plasma from blood followed a protocol described in our previous work [5]. CSF was obtained with a 26-gauge cannula through a 22-gauge guide to lateral ventricles (LV; coordinates of AP − 0.40 mm relative to bregma, ML ±1.50 mm, and DV − 2.0 mm). A volume obtained from the LV was 5 μL, and thus the same volume of sterilized aCSF was added back to the LV. Samples were incubated with 1.0 M perchloric acid for 15 min, and then centrifuged at 25,000×g for 30 min. Supernatants were neutralized with 0.2 μL of 1.0 M NaOH. Finally, 0.2 μL of the neutralized supernatants was used to determine MDMA concentration using HPLC-EC (HTEC-500, Eicom, Japan). MDMA was separated through a mobile phase (0.1 M phosphate buffer at pH 6.0, 500 mg/L 1-decanesulfonic acid, 50 mg/L EDTA, and 1.0% methanol) at a rate of 0.50 ml/min. The detection limit was ~ 1.0 ng/sample (or ~ 10 μM in 0.2 μL CSF). MDMA concentrations in the CSF were estimated by comparison with the external standard (provided by National Institute on Drug Abuse; Rockville, MD). MDMA concentration (moles per liter) was calculated according to the equation: C = m/(MW × V) where C stands for the MDMA concentration, m for the amount of MDMA in grams, MW for MDMA molecular weight, V for CSF volumes in liters.

### Measures for serotonergic injury

Experiment 3 was designed to examine initiation and intensification involving serotonergic injury of rats previously having a mild or severe syndrome. Rats were randomly assigned into one of 5 groups, and *T*_cor_ was determined in response to treatment. Specifically, animals assigned into a CTL-S group were previously treated with 0.9% NaCl × 3 at 2 h intervals, and there was no change in *T*_cor_. Animals assigned into an HD-H^─^ group were previously treated with high binge dose (i.e., 10 mg/kg MDMA × 3 at 2 h intervals) under the normal environment where animals underwent hypothermia (H^─^). Animals in the group HD-H^+^ was previously treated with high binge dose (HD) under the warm environment where animals had had hyperthermia (H^+^) in response to HD. Animals assigned into LH-H^─^ group were previously treated with low binge doses (LH; 2 mg/kg MDMA × 3 at 2 h intervals) under the normal conditions showed hypothermia (H^─^) in response to LD. Lastly, animals assigned into LH-H^+^ group were previously treated with LD under the modified environments (e.g., physical activity on a treadmill at 32 °C) having hyperthermia (H^+^) in response to the LD. Previous studies revealed that serotonergic injury is apparent several days after MDMA administration [10, 12, 19]. In the present study, animals survived 7–14 days after MDMA treatment. During survival time, they returned to home-cages under the normal environmental conditions. The test was carried out with three approaches as follows.

#### Brain 5-HT assay

Seven days after MDMA administration, rats were deeply anesthetized with xylazine (6 mg/kg) combined with ketamine (100 mg/kg) injected intraperitoneally, and the brain rapidly removed. The FCx and hypothalamus were dissected out, weighed and homogenized in acidic solvent (0.1 N perchloric acid, 100 mM EDTA, pH 3.0). After centrifugation (25,000 rpm, 30 min, 4 °C), the supernatant was filtered with a 0.2 μm syringe filter and stored at − 80 °C until the assay. 5-HT was determined using the HPLC-EC method as described above. Data (ng/mg wet tissue) were expressed as mean (± s.e.m).

#### SERT immunohistochemistry

Rats were deeply anesthetized with xylazine (6 mg/kg) combined with ketamine (100 mg/kg), and the brain was removed as previously described [20], and then cut into 40 μm sections. A total of 15 sections (600 μm) were collected from the FCx (AP from + 0.8 mm to + 1.4 mm relative to the bregma), and one of every third section was examined using a DAB-nickel protocol [21]. The primary SERT antibody (PC177L; Calbiochem, San Diego, CA, USA) was diluted at 1:5,000. The secondary antibody was the biotinylated rabbit anti-goat IgG obtained from Vector Laboratories (Burlingame, CA, USA). The avidin–biotin–horseradish peroxidase (HRP) complex was purchased from Vector Laboratories, Burlingame, CA, USA). Histological axon density was analyzed by the ImageJ software (Wayne Rasband, MD, USA). Results were confirmed in 4 rat brains. Data are expressed as mean (± s.e.m).

#### Retrograde axonal tracers

This procedure was used to determine functional integrity of serotonergic axonal retrograde transportation from the FCx to the dorsal raphe nucleus (DRN). After survival for 7 days following SAL or MDMA treatment, 0.5 μL 1% fluorogold (FG; Englewood, CO, USA) was injected bilaterally to the FCx of anesthetized rats with xylazine (4 mg/kg) combined with ketamine (80 mg/kg). In the day 14, animals were again deeply anesthetized with xylazine (6 mg/kg) combined with ketamine (100 mg/kg). The brain was removed, post-fixed and then cut into 40 μm sections. The midbrain sections (AP 0.9 mm – 1.5 mm relative to the lambda) were collected, and every third section was used for the immunohistochemical study. Polyclonal rabbit anti-TpH2 antibody obtained from Thermo Scientific (Rockford, IL, USA; product No. PA1–778) was used at a dilution of 1: 1,000, and Alexa Fluor 594-conjugated goat anti-rabbit IgG from Invitrogen (product No. A11012) at a dilution of 1: 2,000. Numbers of TpH2-positive and FG-positive neurons from a total of 5 sections were counted and expressed as mean (± s.e.m).

### Statistical analysis

Data are expressed as mean ± s.e.m. of 4–7 rats. Animal numbers in a group are based on results of power analysis and efforts to reduce animal numbers used in the study. A two-way ANOVA with repeated measures (StatView 5.0; SAS, NC, USA) was used to determine a statistical difference between saline and MDMA injection, between binge dosing, or between low and high-doses under the same environmental condition. If a significance was found, further analysis was carried out using the *post-hoc* Scheffe test. In some studies, when appropriate, the student t-test was used to assess the difference between saline and drug treatment. The criterion for statistical significance was set at 0.05.

## Results

### Experiment 1: effect of dose on initiating a syndrome

**Fig. 1:**
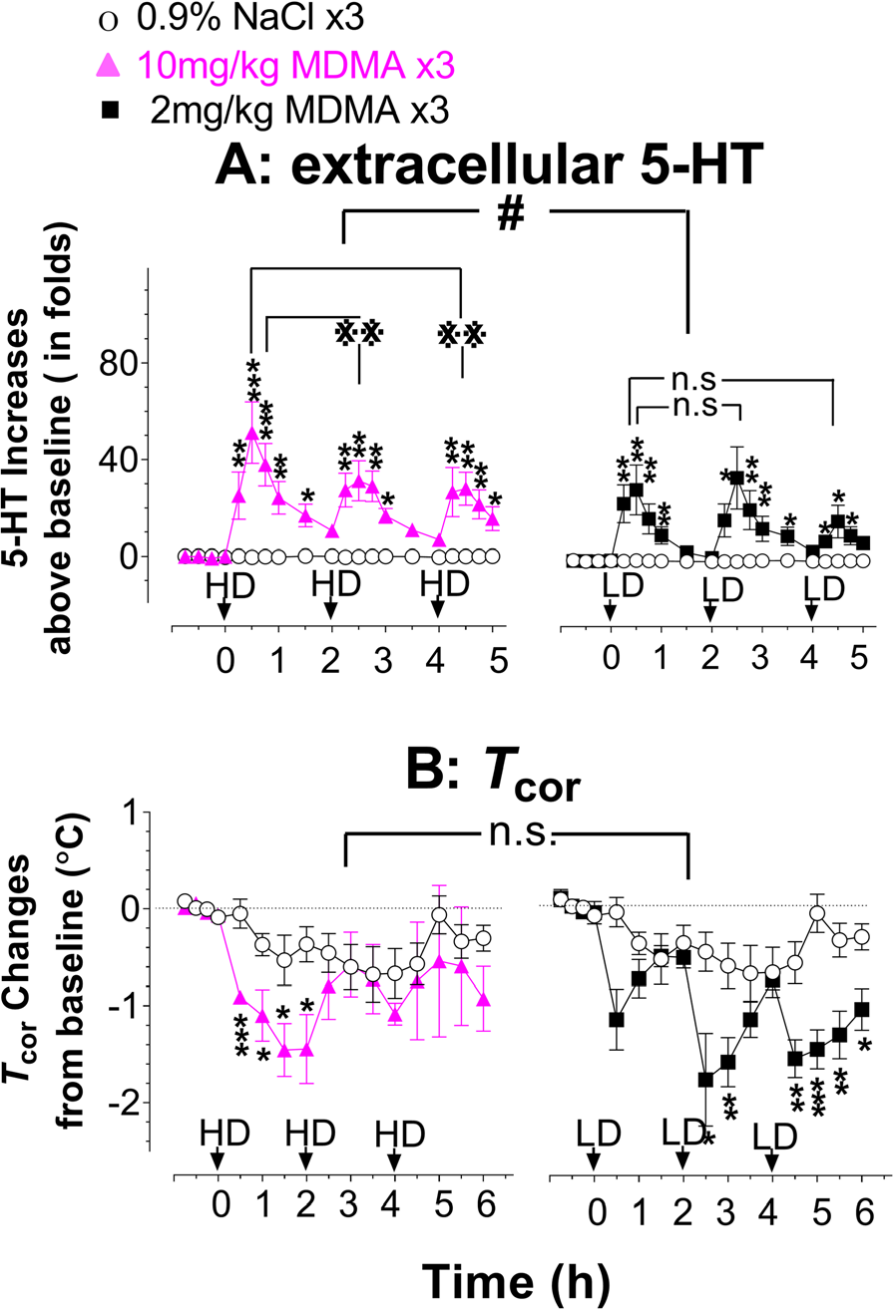
Effect of dose on initiating a syndrome. Occurrence of a syndrome was instrumentally detected with changes in 5-HT and *T*_cor_ under the normal environmental conditions. Arrows indicate the time of binge injection of 10 mg/kg × 3 (high-dose, HD; left panel) or 2 mg/kg × 3 at 2 h intervals (low-dose, LD; right panel). **a** Time course of 5-HT response to MDMA binge injection. X-axis represents time in hours, starting at 0 when given the first drug administration while y-axis represents fold increases in 5-HT above baseline. 5-HT data are expressed as means ± SEM (10 mg/kg × 3, *N* = 6 rats/group; 2 mg/kg × 3, *N* = 5–6 rats/group). *P < 0.05, **P < 0.01 and ***P < 0.001 vs. the SAL group at the same time point. **P < 0.01, indicating the first HD injection vs. the second or third HD injection. n.s., not significant, indicating the first LD injection vs. the second or third LD injection. ^#^*P* < 0.05, indicating the animal group with 10 mg/kg × 3 vs. the group with 2 mg/kg × 3. **b** Time course of *T*_cor_ response to MDMA binge injection. X-axis represents time in hours, starting at 0 as given the first drug administration while y-axis represents changes in *T*_cor_ from baseline (38.54 ± 0.31 °C, *N* = 22). *P < 0.05, and ***P < 0.001 vs. the SAL group at the same time point. n.s., not significant, indicating the animal group with 10 mg/kg × 3 vs. the group with 2 mg/kg × 3

Experiments were conducted under the normal environmental conditions as described in Additional file 1: Figure S1. Effects on syndrome initiation of the high-dose (HD; 10 mg/kg × 3) was compared with the low-dose (LH; 2 mg/kg × 3). First, 5-HT profiles in response to the two doses were compared (Fig. 1a). Basal 5-HT in the FCx was 0.26 ± 0.08 pg/sample (*n* = 23). Compared to SAL × 3, 5-HT significantly increased following the HD (F_(1,10)_ = 41.759, *P* < 0.0001) and the LD (F_(1,9)_ = 9.47, *P* = 0.0132). The maximum increase was over 50- and 30-fold, respectively. In addition, the HD caused a significantly greater increase in 5-HT than the LD (F_(1,9)_ = 6.002, *P* = 0.0368).

Next, effects of the two doses on *T*_cor_ were compared (Fig. 1b). The basal *T*_cor_ was 38.54 ± 0.31 °C (*N* = 22). The HD caused a significant reduction in *T*_cor_ after the first injection (F_(1,9)_ = 10.802, *P* = 0.0094), but not the second (F_(1,9)_ = 0.336, *P* = 0.5766) or third injections (F_(1,9)_ = 0.307, *P* = 0.593). The LD caused a reduction in *T*_cor_ over the 6 h observation period (the first injection, F_(1,9)_ = 3.16, *P* = 0.1082; second injection, F_(1,9)_ = 6.464, *P* = 0.0316; third injection, F_(1,9)_ = 12.195, *P* = 0.0068). However, there was no significant difference between the two doses (F_(1,9)_ = 1.654, *P* = 0.2305).

**Fig. 2:**
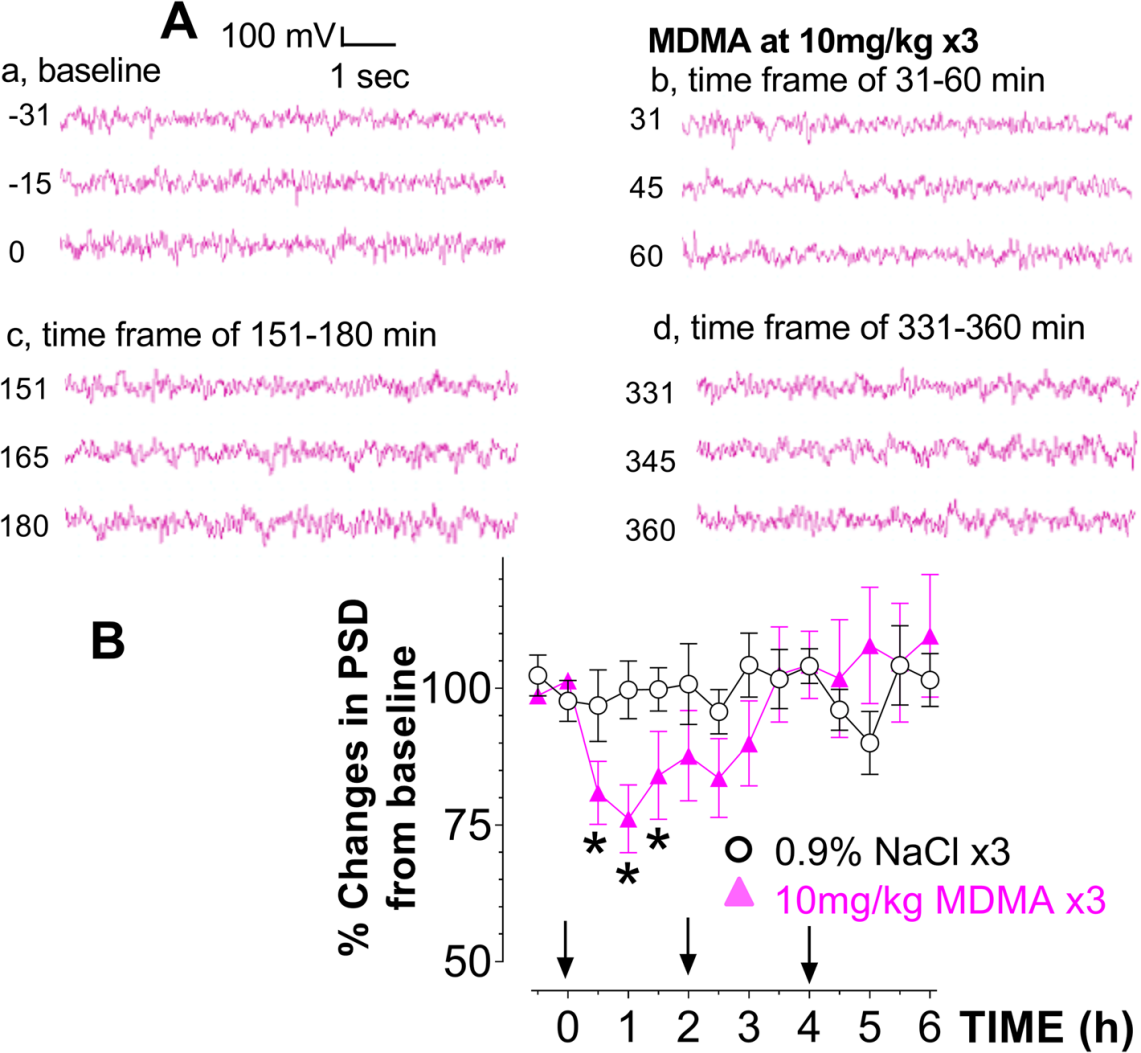
Effect of high-dose (HD, 10 mg/kg × 3) on initiating a syndrome. Occurrence of a syndrome was instrumentally detected with changes in EEG activity under the normal environmental conditions. **A** Representative samples of baseline (a) and the EEG responses to a first (b), second (c) and third injection (d). **B** X-axis represents time in hours, starting at 0 when given the first drug administration while y-axis represents % change in power spectral density (PSD) from baseline (*N* = 6 rats/group). Significant difference from the control group was found in the first injection; F_(1,10)_ = 5.621, *P* = 0.0392), but not the second (F_(1,10)_ = 0.921, *P* = 0.3598) or third injection (F_(1,10)_ = 0.552, *P* = 0.4746). *P < 0.05 vs. the SAL control

**Fig. 3:**
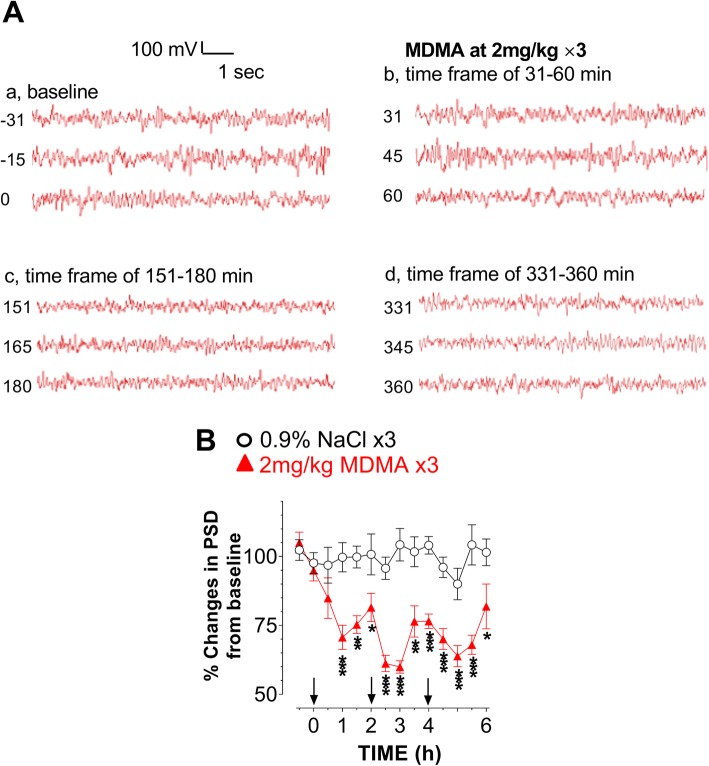
Effect of low-dose (LD, 2 mg/kg × 3) on initiating a syndrome. Occurrence of a syndrome was instrumentally detected with changes in EEG activity under the normal environmental conditions. **A** Representative samples of baseline (a) and the EEG responses to a first (b), second (c) and third injection (d). **B** X-axis represents time in hours, starting at 0 when given the first drug administration while y-axis represents % change in power spectral density (PSD) from baseline. MDMA caused a time-dependent reduction in power density: the first injection, F_(1,10)_ = 14.561, *P* = 0.0034; second injection, F_(1,10)_ = 52.313, *P* < 0.0001; third injection, F_(1,10)_ = 30.072, *P* = 0.0003. *P < 0.05, **P < 0.01 and ***P < 0.001 vs. the SAL control

Lastly, we compared the EEG profile in response to the two doses. Compared to respective baseline, EEG amplitudes appeared to be reduced following the HD (Fig. 2A) and the LD (Fig. 3A). These amplitudes were digitally quantified and expressed as power spectral density (PSD). Compared to respective PSD baseline, there was a significant reduction in PSD or desynchronization following the HD (Fig. 2B) and the LD (Fig. 3B).

### Experiment 2: effect of environment on intensifying syndrome intensity

**Fig. 4:**
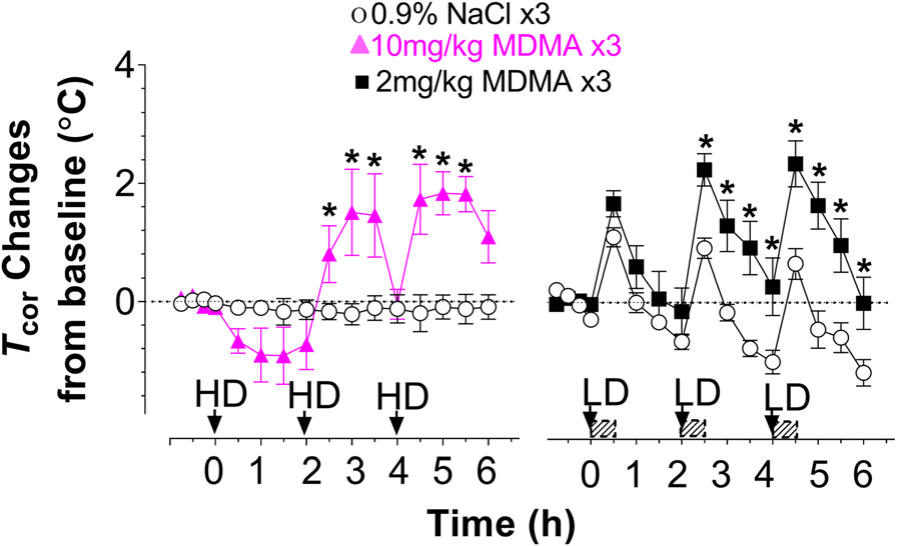
Effect of environment on the syndrome intensification. The syndrome intensity was estimated with changes in *T*_cor_ under the modified environmental conditions. X-axis represents time in hours, starting at 0 as given the first drug administration of HD (left panel) or LD MDMA (right panel) while y-axis represents changes in *T*_cor_ from baseline Arrows indicate the time of binge injections. The red horizontal bars indicate the time period (30 min) of treadmill exercise.10 mg/kg × 3, *N* = 6 rats/group; 2 mg/kg × 3, *N* = 6 rats/group. MDMA no longer caused a reduction in *T*_cor_. Instead, there were increases in *T*_cor_ (hyperthermia) *P < 0.05 vs. the SAL group of the same time point). *P < 0.05, **P < 0.01 and ***P < 0.001 vs. the SAL group of the same time point)

Hyperthermia is the major concern in MDMA abuse. However, MDMA under the normal condition can only cause a reduction in *T*_cor_, or hypothermia. Previous studies showed that hyperthermia occurs only under the modified environment [10, 12, 19, 22]. However, this effect was examined mainly with the high binge dose, rarely the low binge dose. In the following experiments, we tested that 1) hyperthermia indicative of severe syndrome could be elicited not only by the high binge dose, but also the low binge dose; 2) mechanism underlying the syndrome intensification was not attributed to the extracellular 5-HT in the brain. Details of modified environment in accord with drug doses described under the Methods section and Additional file 1: Figure S1. First, animals were placed in the modified environment for habituation for 2 h prior to *T*_cor_ measurement. *T*_cor_ prior to drug administration was 38.84 ± 0.51 °C for the HD group (*N* = 11), and 38.48 ± 0.27 °C for the LD group (*N* = 13). Next, drugs were administered to the respective groups. As shown in Fig. 4, the HD did not produce significant change in *T*_cor_ following the first injection (F_(1,9)_ = 2.608, *P* = 0.1408). However, hyperthermia developed after the second (F_(1,9)_ = 6.571, *P* = 0.0305) and third injections (F_(1,9)_ = 31.647, *P* = 0.0003). Similarly, The LD had no significant effect on the *T*_cor_ following the first injection (F_(1,10)_ = 3.822, *P* = 0.0791), but the *T*_cor_ significantly elevated after the second injection (F_(1,10)_ = 21.67, *P* = 0.0009) and third injection (F_(1,10)_ = 14.418, *P* = 0.0035).

**Fig. 5:**
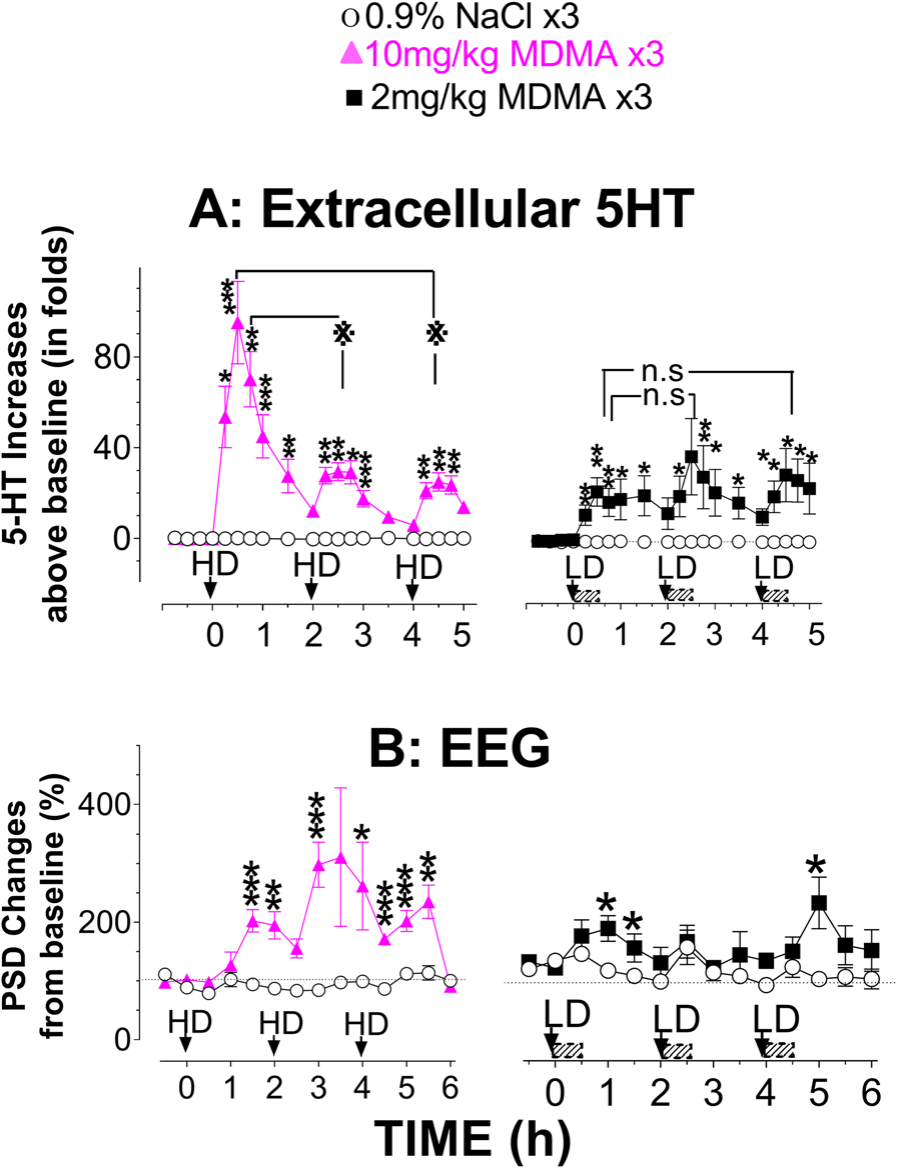
Effect of environment on the syndrome intensification. The syndrome intensity was estimated with changes in 5-HT (**a**) and EEG (**b**) under the modified environmental conditions. **a** Time-course of *T*_cor_ response to binge injection of HD (left panel) or LD MDMA (right). X-axis represents time in hours, starting at 0 when given the first drug administration while y-axis represents fold increases in 5-HT above baseline. *P < 0.05, **P < 0.01 and ***P < 0.001 vs. the SAL group at the same time point. **P < 0.01, indicating the first HD injection vs. the second or third HD injection. n.s., not significant, indicating the first LD injection vs. the second or third LD injection. **b** Time-course of EEG response to binge injection of HD (left panel) or LD MDMA (right). X-axis represents time in hours, starting at 0 as given the first drug administration while y-axis represents % change in power spectral density (PSD) from baseline. 10 mg/kg × 3, *N* = 4–6 rats/group; 2 mg/kg × 3, *N* = 4–6 rats/group. MDMA no longer caused a reduction in PSD (or desynchronization). Instead, there were increases in PSD (synchronization) under enriched environmental conditions

Figure 5a displays changes in 5-HT following the HD or the LD in separated sets of animals under modified environments. Compared to respective SAL control, 5-HT was significantly increased after the HD (F_(1,10)_ = 22.847, *P* = 0.0007; and LD, F_(1,10)_ = 9.812, *P* = 0.0106). The maximum increase was nearly 90-fold and 20-fold, for the HD and LD, respectively. We then tested whether brain activity was altered differently as the syndrome severity was intensified from the mild to severe levels. Figure 5b demonstrates the percentage changes in PSD. EEG activity increased by both the HD [the first dose, F_(1,8)_ = 23.562, *P* = 0.0013; second, F_(1,8)_ = 15.131, *P* = 0.00046; third, F_(1,8)_ = 41.488, *P* = 0.0002), and the LD [first, F_(1,8)_ = 7.98, *P* = 0.0223; second, F_(1,8)_ = 0.96, *P* = 0.3558; third, F_(1,8)_ = 4.615, *P* = 0.044].

MDMA concentrations in the CSF and plasma were measured, 30 min after administration. The HD produced ~ 20–50 μM MDMA in the CSF and ~ 20–58 μM in the plasma (Table 1). There was no difference between the three injections (*P* > 0.05; t-test), or between the environmental conditions (*P* > 0.05; t-test). However, following the LD, MDMA was not detectable in either the CSF or plasma with our HPLC-EC.

**Table 1:** MDMA concentrations (μM; *N* = 3) in the CSF and plasma

	CSF		Plasma	
	22 °C	26 °C	22 °C	26 °C
First HD	39.9 ± 21.3	20.2 ± 3.0	38.0 ± 23.7	19.8 ± 2.4
Second HD	48.5 ± 21.8	35.0 ± 12.8	57.8 ± 33.0	27.6 ± 11.4
Third HD	48.5 ± 17.7	21.1 ± 18.5	44.3 ± 21.6	32.3 ± 10.0

Samples were taken 30 min after HD (10 mg/kg) injection

### Experiment 3: effect of dose and environment, respectively, on initiating and intensifying serotonergic injury in rats previously having a mild or severe syndrome caused by MDMA

**Fig. 6:**
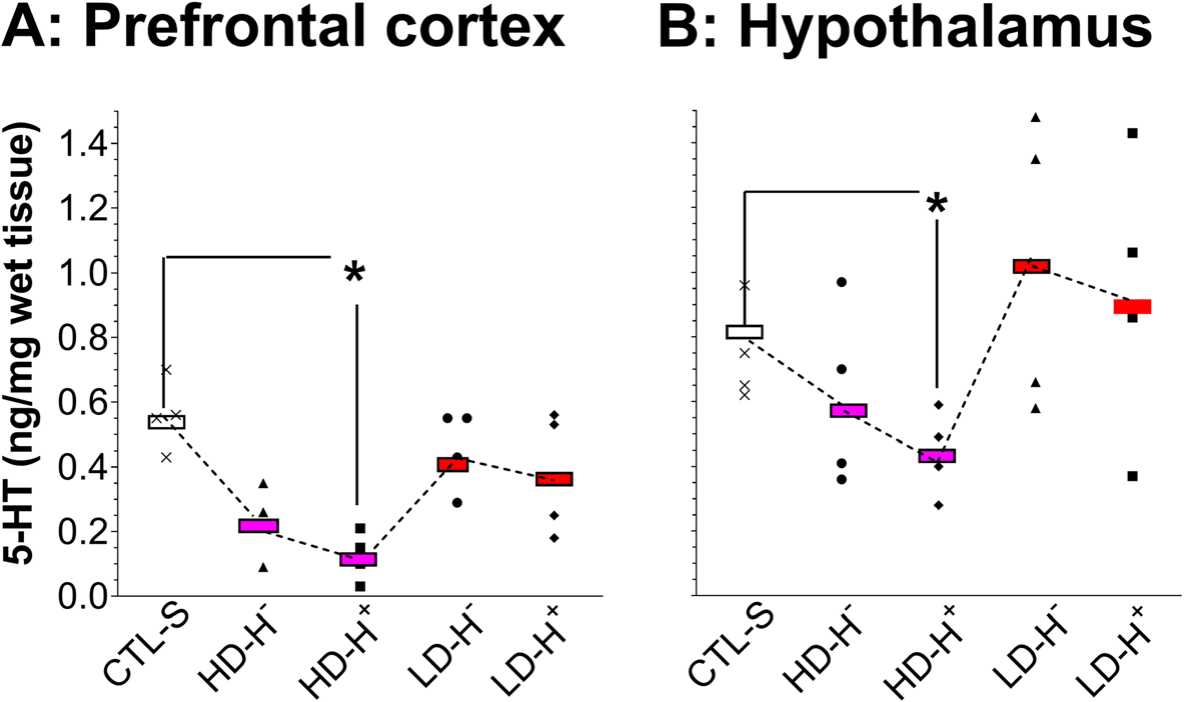
Effects of MDMA doses and environments, respectively, on initiating and intensifying serotonergic injury. Tissue 5-HT content was used to estimate those effects. Data are expressed as an individual level (ng/mg wet tissue) for each treatment group (*N* = 4–6). **a** Changes in the FCx. **b** Changes in the hypothalamus. *P < 0.05 vs. the SAL group. *CTL-S*, saline as control; *HD-H* ^*─*^, high-dose-induced hypothermia; *HD-H*^*+*^, high-dose-induced hyperthermia; *LD-H* ^*─*^, low-dose-induced hypothermia; *LD-H*^***+***^, low-dose-induced hyperthermia

After survival from a syndrome for 7 days, animals previously having hypothermia (H^─^) as the mild syndrome or hyperthermia (H^+^) as the severe syndrome were sacrificed and tissue 5-HT was evaluated for injury. In rats with the HD-induced hypothermia (HD-H^─^ group), there was a tendency of 5-HT reduction, but it was not statistically significant compared to the CTL-S (Fig. 6). In the brain tissue from HD-hyperthermia (HD-H^+^) group, the reduction was statistically significant in the FCx (Fig. 6a), and hypothalamus (Fig. 6b). In the LD-H^+^ group, there was no significant reduction in brain tissue 5-HT, despite having hyperthermia previously. Tissue 5-HT from the LD-H^─^ group was not different from the CTL-S group. To reduce the number of animals used in the study, the LD-H^─^ group was omitted from the rest of following experiments.

**Fig. 7:**
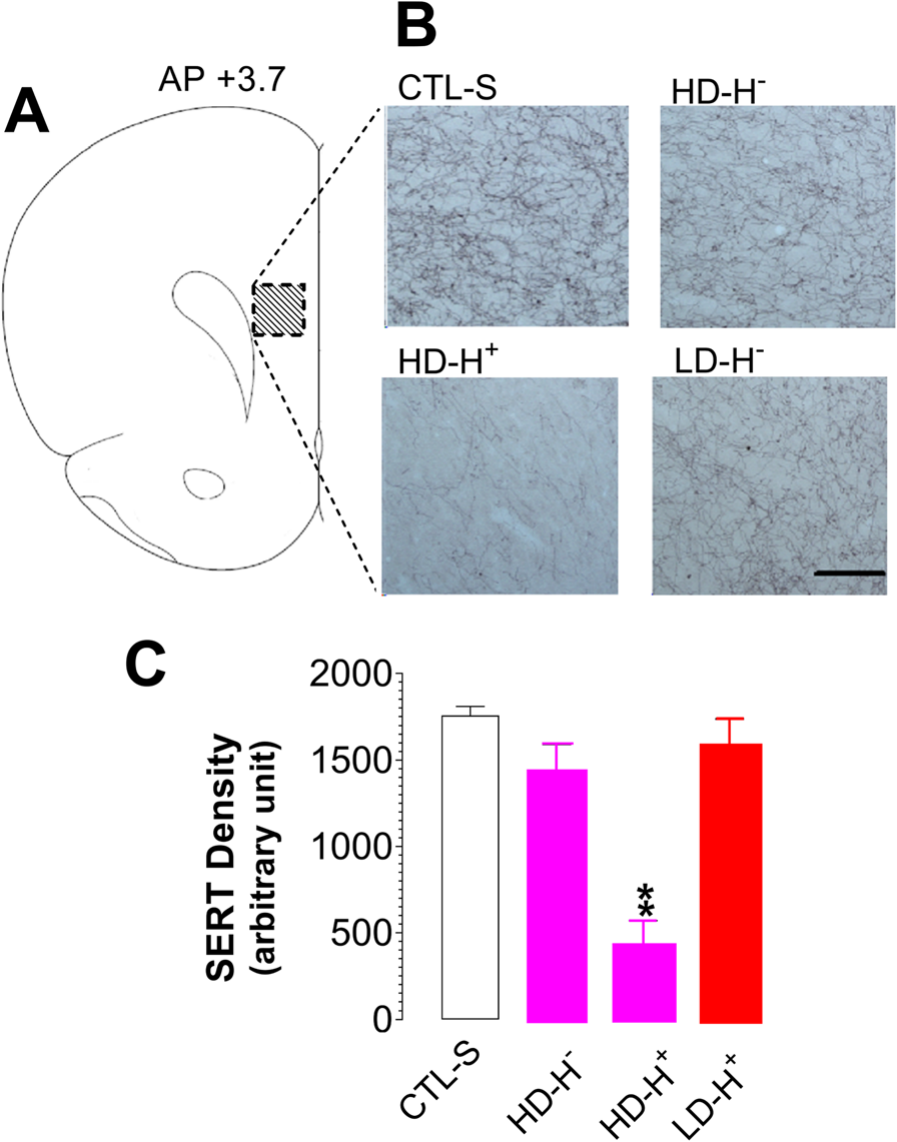
Effects of MDMA doses and environments, respectively, on initiating and intensifying serotonergic injury. SERT density was used to estimate those effects. **a** Schematic diagram of the medial FCx and corresponding coronal sections used to assess the SERT-containing fiber density (anterior-posterior location + 3.70 relative to bregma). **b** Changes in SERT-containing fibers. Bar: 100 μm. **c** The means of optical densities (arbitrary unit). **P < 0.01 vs. CTL, examined with ANOVA followed by post-hoc test. *CTL-S*, saline as control; *HD-H* ^*─*^, high-dose-induced hypothermia; *HD-H*^*+*^, high-dose-induced hyperthermia; *LD-H*^***+***^, low-dose-induced hyperthermia

Figure 7a and b show representative microphotographs of SERT immunoreactivity in the FCx. Except for the HD-H^+^ group, there was no visible change in SERT fiber density. The fiber density in the HD-H^+^ group became sparse. Compared to CTL-S, the density in the HD-H^+^ group was significantly reduced (Fig. 7c; F_(3,13)_ = 16.16, *P* = 0.0001).

**Fig. 8:**
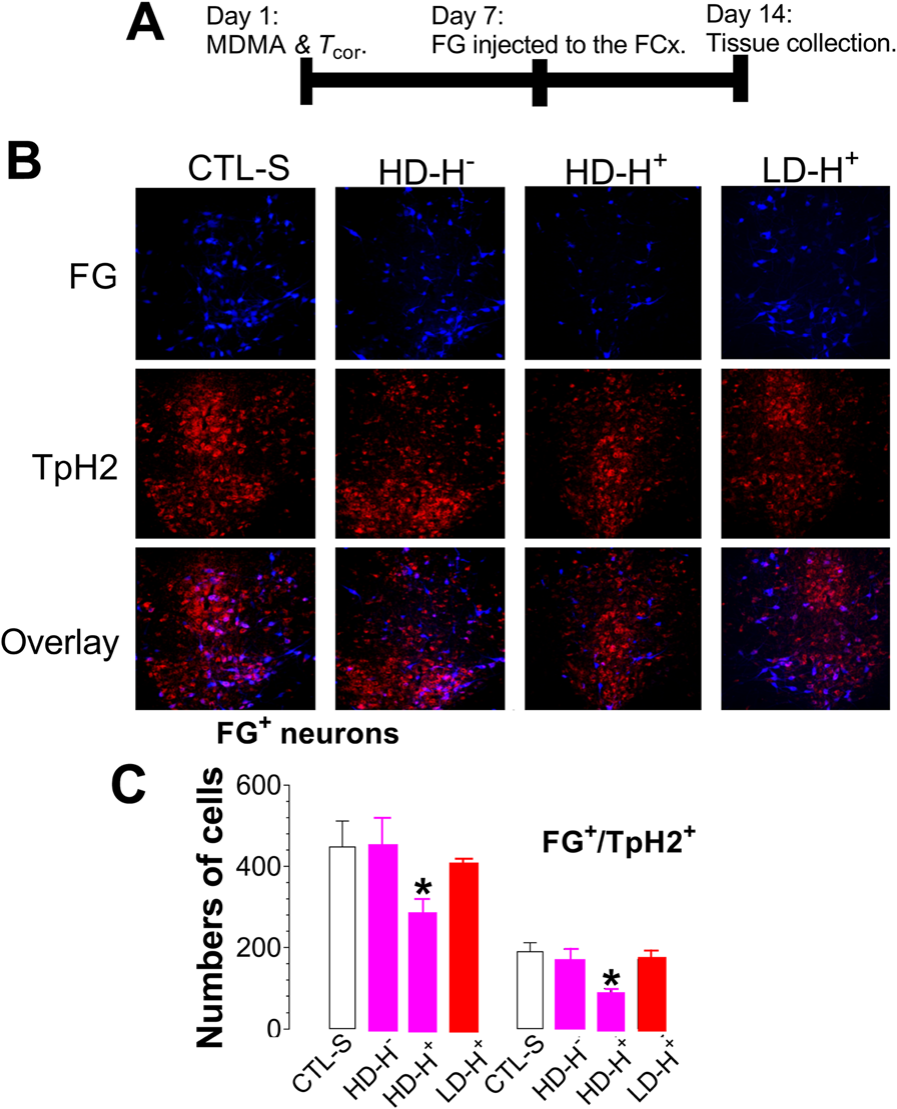
Effects of MDMA doses and environments, respectively, on initiating and intensifying serotonergic injury. Serotonergic axonal retrograde transportation was used to estimate those effects. **a** Timeline of MDMA or SAL treatment followed by *T*_cor_ measurement (day 1), fluorogold administration (FG; day 7) and tissue sectioning (day 14). **b** Immunohistochemical staining of tryptophan hydroxylase-2 (TpH2)-containing neurons in the DRN retrograded with FG. Photomicrographs show representative changes in FG-positive (FG^+^) or/and TpH2-positive (TpH2^+^) neurons in the DRN. The top row, the FG^+^ neurons; the middle row, the TpH2^+^ neurons; the bottom row, FG^+^/TpH2^+^. **c** Cell counts of FG^+^ and FG^+^/TpH2^+^ neurons (*N* = 4–6). **P < 0.01 vs. the SAL control, examined using unpaired t-test. *CTL-S*, saline as control; *HD-H* ^*─*^, high-dose-induced hypothermia; *HD-H*^*+*^, high-dose-induced hyperthermia; *LD-H*^***+***^, low-dose-induced hyperthermia

Lastly, we examined whether the functional integrity of serotonergic axonal retrograde transportation from the FCx to the dorsal raphe nucleus (DRN) was impaired in rats previously having MDMA toxicity. Figure 8a shows the timeline for MDMA and *T*_cor_ measurement (day 1), the retrograde tracer fluorogold (FG) microinjection to the FCx (day 7), and immunocytochemical staining of the DRN neurons (day14). Numbers of cells were counted and then classified as TpH2-containing (Fig. 8b; TpH2^+^), FG-containing (FG^+^), and TpH2/FG-containing neurons (FG^+^/TpH2^+^). Except for the HD-H^+^ group, changes in *T*_cor_ in the acute effect did not have an impact on the retrograde transportation of FG to the DRN or to the TpH2-containing neurons. Noticeably in the HD-H^+^ group, there was a 40% reduction in FG retrograde transportation to TpH2-containing neurons (*p* < 0.05; Fig. 8c).

## Discussion

The results of the present study clearly indicate that a toxicity course showing initiation followed by intensification demonstrated at the serotonin syndrome in our previous work [3, 6] is also found in the development of serotonergic injury. However, the molecules mediating the serotonin syndrome may be distinctly different from those for processing serotonergic injury.

In a serotonin syndrome, it is obvious that the excessive increase in extracellular 5-HT is the cause of this kind of toxic effect. However, 5-HT levels are found to have no proportional relationship with hypothermia in the mild or hyperthermia in the severe serotonin syndrome ([3]; also in this study). This suggests that molecules for the syndrome initiation are different from those for intensification, and that intensification cannot be simply achieved by increasing drug doses, or specifically, brain 5-HT levels. It is well documented that hypothermia and hyperthermia can be evoked by activation of 5-HT_1A_ and 5-HT_2A_ receptors, respectively in the hypothalamus [23, 24]. We found that 5-HT was also excessively elevated in the hypothalamus (Additional file 1: Figure S2), consistent with possible thermoregulation involving two distinct receptors. Other receptors such as dopamine D_1_, D_2_, and adrenergic receptors could also be involved in thermoregulation [11, 25], which are beyond the scope of this research and not further discussed. It has been suggested that 5-HT_1A_ receptor-mediated hypothermia is neuroprotective [26] and likely prevent harmful actions or further deterioration. Thus, it is not surprising to see that mild serotonin syndrome is not life-threatening, which can be self-abolished as time passes by. It appears that 5-HT has high affinity to 5-HT_1A_ receptors [27, 28], and thus usually leading to hypothermia to terminate the toxic deterioration.

Activity balance between 5-HT_1A_ and 5-HT_2A_ receptors can be altered in adaptation to the drug environment. At warm temperatures, activity of 5-HT_1A_ receptors is tempered [29] while activity of 5-HT_2A_ receptors is exaggerated [30]. Thus, it is likely that drug environment causes reduced responsiveness of 5-HT_1A_ receptors and enhanced responsiveness of 5-HT_2A_ receptors to 5-HT in the thermoregulatory pathways, which may be a potential mechanism underlying hyperthermia and severe serotonin syndrome under the modified environment. Given the fact that Ecstasy abuse is more likely concomitant with a strenuous dance, physical activity should be considered to be another important factor that affects the activity balance of two receptors, which may be of worthwhile investigation in the future.

Analysis of EEG has been considered a powerful approach to reveal the psychobiological changes in response to drug abuse [31, 32]. EEG waves are derived from cortical networks reflecting the dynamic integration between excitatory and inhibitory synaptic potentials [33, 34]. EEG waves are usually categorized at least into delta, theta, alpha and beta bands based on amplitudes and oscillation frequencies. We found that these bands had the same tendency in response to MDMA and environmental changes, and thus our data presentation focused on the overall changes in the power spectral density (PSD) of EEG waves, rather not on a specific band. The advantage of the PSD study is that it could be readily used to estimate EEG desynchronization and synchronization. It has been suggested that EEG desynchronization may be related to arousal [34, 35], while synchronization may indicate psychobiological impairment or drowsiness [36]. We found that the LD, similar to the HD, caused EEG desynchronization under the normal environment, consistent with the suggestion that MDMA similar to other amphetamine-like drugs usually causes wakefulness [37]. It is noteworthy that the modified environment reversed the EEG response from MDMA-induced desynchronization to synchronization. EEG synchronization is commonly present in sleep, but also drowsiness [38], hallucinations [39], or seizures [40]. Although few clinical EEG data are available, patients with severe MDMA toxicity are known to have disorientation, hallucinations or seizures [41, 42]. It has been suggested 5-HT_1A_ and 5-HT_2A_ receptors exert opposite effects on cortical pyramidal neurons associated with EEG activity [43]. Taken together, EEG findings in line with the *T*_cor_ and neurochemical data demonstrated that there are aberrant EEG powers in the serotonin syndrome, supporting the suggestion that environmental conditions have impacts on the activity balance between 5-HT_1A_ and 5-HT_2A_ receptors in adaptation to the drug environment associated with the syndrome intensity in MDMA toxicity.

Results of the present study support that serotonergic injury is also an array of toxic initiation and intensification. Consistent with the hypothesis, our findings that tissue 5-HT was slightly reduced in the HD-H^─^ group and apparent in the HD-H^+^ but not LD-H^─^ or LD-H^+^ groups (see details in Fig. 6) suggest that the injury initiation is likely associated with the HD. The cause of injury is likely ascribed to a high concentration of MDMA itself, but unlikely MDMA-elicited increase in 5-HT. MDMA-elicited cell death is well documented in literature, including those with Petri dish studies at the MDMA concentrations exceeding 200 μM [44]. In the present study, we found that systemic administration of MDMA caused a reduction in FG^+^ but not TpH2^+^. This suggests that, despite functional impairment, there was no cell death in line with previous findings [45]. One possible explanation for the discrepancy between in-vivo and in-vitro studies is that MDMA concentration was much lower in the brain than that examined in Petri dishes. MDMA is widely distributed in the brain after administration [46]. Our results showed that MDMA concentration in the CSF was less than ~ 50 μM following the HD, far smaller than 200 μM that presumably causes cell death in vitro. We found that MDMA concentrations in the CSF were closely similar to those in the plasma, suggesting that plasma can be used to estimate MDMA concentrations in the CSF. It was estimated previously that plasma MDMA was only ~ 10 μM [18, 47], or maximally 100 μM [48]. Our plasma results are consistent with those reports [18, 47, 49] which revealed that the amount of MDMA distributed in the brain after injection of the HD was much less than the concentration that would cause cell death in vitro. A drawback in our MDMA assay, however, is that MDMA was not detectable at the LD. We found that MDMA at 10 μM and below could cause an acute increase in 5-HT exceeding 10-fold above baseline (Additional file 1: Figure S3), also supporting the suggestion that MDMA concentrations required for a serotonin syndrome are much lower than that for the brain injury.

We found that injury was apparent in the HD-H^+^ but not HD-H^─^ groups. Since H^+^ represents modified environment, this is consistent with the proposal that environments are critical for intensifying serotonergic injury. However, although environments have an impact on the activity balance between 5-HT_1A_ and 5-HT_2A_ receptors as discussed above, those mechanisms are unlikely to contribute to serotonergic injury. It has been suggested that MDMA at the concentration of 10 μM and higher was sufficient to cause mitochondrial dysfunction, producing reactive oxygen species (ROS; [44, 49]). It is likely that the MDMA concentration following the LD was far below the threshold level for changing mitochondrial function, which is worthy of further investigation. Furthermore, the ROS generation relied on *T*_cor_ [50], suggesting an environmental-dependent manner. Increasing ROS was likely responsible for serotonergic injury [51]. Taken together, the injury intensification is likely associated with the ROS generation but further experimental confirmation is warranted.

## Conclusion

We found that the low-dose was sufficient to initiate a serotonin syndrome due to excessive increases in extracellular 5-HT that exerts postsynaptic effects on 5-HT_1A_ and 5-HT_2A_ receptors. In contrast, the high-dose is needed to generate the ROS for the serotonergic injury due to the impairment of mitochondrial function, suggesting that MDMA concentration in the brain is the determinant for toxic initiation and reactive oxygen species generation for intensification. Thus, the courses of two adverse effects are totally different in the toxic initiation and intensification, which may explain the observation that a serotonin syndrome is not always followed by serotonergic injury.

## Additional file

Additional file 1: **Figure S1.** Experimental designs. **Figure S2.** Effects of the modified environment on 2mg/kg MDMA-elicited increases in hypothalamic 5-HT. **Figure S3.** Correlation between 5-HT elevation and MDMA concentrations in the FCx. (DOCX 203 kb)

## Data Availability

All data have been available from the corresponding author upon request.

## Abbreviations

5-HT: 5-hydroxytryptamine
CSF: Cerebrospinal fluid
CTL: Control
DA: Dopamine
DAB: Diaminobenzidine
DRN: Dorsal raphe nucleus
EEG: Electroencephalogram
FCx: Prefrontal cortex
FG: Fluorogold
H^−^: Hypothermia
H^+^: Hyperthermia
HD: High-dose
LD: Low-dose
MDMA: 3,4-methylenedioxy-N-methylamphetamine
PSD: Power spectral density
ROS: Reactive oxygen species
SERT: Serotonin transporter
TpH2: Tryptophan hydroxylase-2
