# Supplementary material for: Differential role of dose and environment in initiating and intensifying neurotoxicity caused by MDMA in rats
Source: BMC Pharmacol Toxicol. 2019 Aug 5;20:47. doi: 10.1186/s40360-019-0326-6 (PMC6683525; doi:10.1186/s40360-019-0326-6)
Supplement: Supplementary file 1 — Figure S1. Experimental designs. Figure S2. Effects of the modified environment on 2mg/kg MDMA-elicited increases in hypothalamic 5-HT. Figure S3. Correlation between 5-HT elevation and MDMA concentrations in the FCx. (DOCX 203 kb) [file 40360_2019_326_MOESM1_ESM.docx]

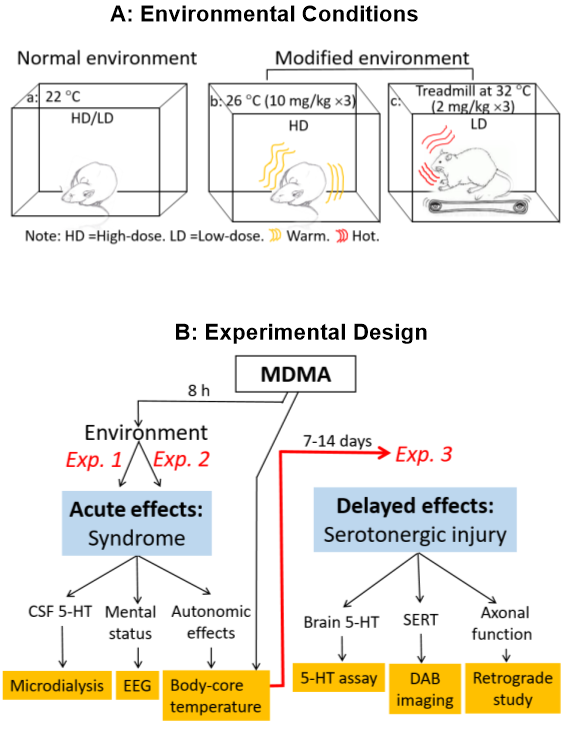


**Figure S1 Experimental designs**. **A**, Environmental conditions. Plexiglas was used to construct a chamber with a ventilation system providing a constant airflow to the chamber linked to refrigeration or heater units. The chamber (L 100 cm × W 60 cm × H 80 cm) was large enough to hold a Raturn or animal activity cage (BASi, West Lafayette, IN, USA) or treadmill apparatus. Environmental temperature was automatically regulated through a temperature regulator, accurate to an environmental temperature ±1 °C. MDMA at 2mg/kg ×3 at 2h intervals was defined as the low-dose (LD) and 10mg/kg ×3 as the high-dose (HD). Experiment conducted at 22 °C was defined as the normal environment. Chamber temperature was set at 26 °C as the modified environment for testing HD rats, and at 32 °C in combination of a treadmill for testing LD rats. **B**, Methods to estimate two aspects of MDMA toxicity. Intensity of serotonin syndrome was determined by measuring extracellular 5-HT with microdialysis, brain’s electrical activity with EEG, and changes in body-core temperature (*T*_cor_). Serotonergic injury was estimated by measuring brain 5-HT content, SERT and axonal retrograde transportation, 7-14 days after a syndrome


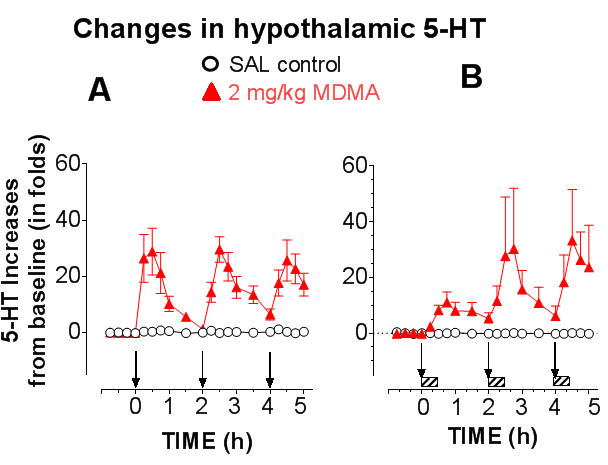


**Figure S2 Effects of the modified environment on 2mg/kg MDMA-elicited increases in hypothalamic 5-HT.** Arrows indicate the time of injections at 2h intervals. N =4-6 rats/group **A**, Rats were freely behaving at 22 °C (±1 °C). **B**, Animals placed on a treadmill at a warm temperature of 32 °C (±1 °C). The red horizontal bars indicate the time period (30 min) of treadmill exercise. The enriched environment had no effect on MDMA-elicited 5-HT in the hypothalamus.

**Methods:** Stereotaxic coordinates for hypothalamic guide cannulas (10mm in length of an 22 gauge stainless steel tubing) were, AP -1.80 mm relative to bregma, ML 0.90 mm to midline, and DV -2.0 relative to the skull surface. A microdialysis probe was a 2.5 mm hollow nitrocellulose fiber (200μm i.d., 13,000 MW cut-off; Spectrum Medical Industries, Los Angeles, CA, USA). The probe was implanted into the hypothalamus through the guide cannulas, targeting to AP -1.80 mm relative to the bregma, ML 0.90 mm relative to the midline and DV 7.0-9.50 mm relative to the skull surface (see details in Supplemental S1).


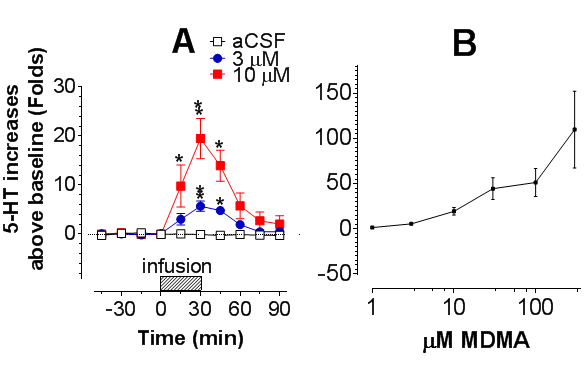


**Figure S3.** **Correlation between 5HT elevation and MDMA concentrations in the FCx.** **A**, Time course of 5HT response to 3 µM and 10 µM MDMA in the microdialysis probe. Horizontal bars indicate the time of MDMA infusion. *P <0.05 and **P <0.01 *vs.* respective time points of vehicle control. **B**, Concentration-dependent effects of MDMA on 5HT elevation. N =4-6 rats/group. Note that the delivery efficiency of MDMA was 21.9% (±11.6; N =3) in test tubes at room temperature of 22 °C (*N* =3). However, MDMA concentrations in the graphs are not corrected from the probe delivery efficiency.

**Methods:** MDMA was dissolved in the aCSF for a concentration of 3 µM or 10 µM, and then delivered through a probe in the FCx. With the same probe, extracellular 5-HT was obtained from the same region.
